# Interaction Effect of RsaI and BamHI Polymorphisms of TGFα, BMP2 and BMP4 on the Occurrence of Non-Syndromic Cleft Lip and Palate in Iranian Patients

**Published:** 2018

**Authors:** Saba Samadi, Asghar Ebadifar, Hamid Reza Khorram Khorshid, Koorosh Kamali, Mohammadreza Badiee

**Affiliations:** 1. General Dentist, Tehran, Iran; 2. Dentofacial Deformities Research Center, Research Institute of Dental Sciences, Department of Orthodontic, Faculty of Dentistry, Shahid Behehsti University of Medical Sciences, Tehran, Iran; 3. Genetic Research Centre, University of Social Welfare and Rehabilitation Sciences, Tehran, Iran; 4. Department of Public Health, Faculty of Public Health, Zanjan University of Medical Sciences, Zanjan, Iran; 5. Dentofacial Deformities Research Center, Research Institute of Dental Sciences, Shahid Beheshti University of Medical Sciences, Tehran, Iran

**Keywords:** Bone morphogenetic proteins, Cleft Lip and palate, Polymorphism

## Abstract

**Background:**

Orofacial cleft is the most common congenital defect of the maxillofacial region. Its non-syndromic type is multi-factorial, and several genes are involved in its occurrence. This study aimed to assess the interaction effect of Rsal and BamHI polymorphisms of *Transforming Growth Factor-alpha (TGFα)* gene and Bone Morphogenetic Protein-2 (BMP2) and BMP4 variants on the occurrence of Non-Syndromic Cleft Lip and Palate (NSCLP) in the Iranian population.

**Methods:**

This case-control study was conducted on 120 children with NSCLP and 215 healthy children. Genotyping of the TGFA/BamHI (rs11466297), TGFA/RsaI (rs37322-48), BMP4 (rs17563) and BMP2 (rs235768) was performed by Polymerase Chain Reaction (PCR) and Restriction Fragment Length Polymorphism (RFLP) methods. Logistic regression was applied to determine the effective factors and the interaction effect of different variants on the occurrence of NSCLP.

**Results:**

Gender of patients had no significant association with the occurrence of NSCLP (p=0.335). Multiple logistic regression showed that the interaction effect of the aforementioned polymorphisms on the occurrence of NSCLP was not statistically significant (p=1.000).

**Conclusion:**

Although the individual effect of each of the BMP4, BMP2, RsaI and BamHI variants on the occurrence of NSCLP in the Iranian population has been previously confirmed, their interaction does not play a role in this respect.

## Introduction

Orofacial cleft is the most common congenital defect of the maxillofacial region. It has the highest prevalence in the East Asia such as China and Japan with 1/500 live births and the least prevalence among the African-Americans with 0.21 to 0.41/1000 live births 1,2. The prevalence of orofacial cleft in Iran is 1.05 to 1.9/1000 live births 3–5. Orofacial cleft decreases the quality of life of patients and their family and negatively affects the speech, oral health and mental health of patients 6. The non-syndromic form is multifactorial, and environmental factors such as cigarette smoking, alcohol consumption, folate consumption, infections and viruses as well as genetics may be involved in its occurrence 1,7. Nonetheless, the molecular mechanism of action of these factors has not been well recognized. Several genes may be involved in the occurrence of Cleft Lip and Palate (CLP). TGFα is a member of the epidermal growth factor superfamily, which codes a protein with the same name. During craniofacial development, TGFα is expressed in the internal border of the epithelium of fusing palatal shelves and stimulates the synthesis of extracellular matrix and migration of mesenchymal cells, which further strengthens the palatal tissue. RsaI (rs3732288) and BamHI (rs11466297) are the two common variants of this gene 8,9. Ardinger *et al* evaluated the role of *Transforming Growth Factor-alpha (TGFα)* gene variants in the occurrence of CLP 10. Ebadifar *et al* showed that polymorphism of TGFα variants plays a role in the occurrence of CLP 11.

Evidence shows that Bone Morphogenetic Proteins (BMPs) and their antagonist, Noggin, play a role in fusion of the upper lip and the primary palate 12. The two subgroups of BMP2 and BMP4 play a fundamental role in craniofacial development and are specifically expressed in epithelial and mesenchymal cells of the palatal shelves. The significance of BMP2 (rs235768) and BMP4 (rs17563) variants in the occurrence of CLP has been emphasized in previous studies 13,14. Saket *et al* indicated that polymorphism of BMP2 (rs235768) and BMP4 (rs17563) variants plays an important role in the occurrence of CLP in the Iranian population 15. Blanco *et al* demonstrated that although the interaction between BMP4 and IRF6 is mild, presence of specific haplotypes indicates higher risk of occurrence of CLP 16.

Molecular studies have shown that interaction of gene-gene such as Sonic Hedgehog (SHH), bone morphogenetic proteins, the homeobox containing genes *Barx1* and *Msx1* can control the initiation, outgrowth and specification of the facial processes 17–19. Evidence suggests genetic predisposition of NSCLP, caused by the interactions of multiple interacting genes 20. Song *et al* showed that the interaction of PAX9 and IRF6 plays a potentially important role in the occurrence of CLP 21. Lidral *et al* found that the 4-2-2-2-2 haplotype for MSX1 CA-X1.1-X1.3-X2.1-X2.4 was most frequently transmitted among both CLP and CP cases 22. Jugessur *et al* raised the possibility of interaction between TGFA, TGFB3, and MSX1. The effect of this TGFA TaqI genotype was stronger among children homozygous for the MSX1-CA A4 allele, raising the possibility of interaction between these two genes 23. Since the individual role of polymorphism of the aforementioned variants has been previously confirmed in the occurrence of CLP in the Iranian population 11,15, this study aimed to assess the interaction effect of Rsal and BamHI polymorphisms of TGFα and BMP2 and BMP4 on the occurrence of Non-Syndromic Cleft Lip and Palate (NSCLP) in the Iranian population.

## Materials and Methods

This case-control study was approved in the ethics committee of Shahid Beheshti University of Medical Sciences (IR.SBMU.RIDS.REC.1395.195). The study group included blood samples of 120 children with NSCLP born at Mofid Hospital from 2013 to 2016. Patients with other skeletal and facial malformations such as congenital lip pits, cardiac diseases or neurologic anomalies were excluded. The control group included 215 healthy children born in Tehran city between 2013 to 2016. Written informed consent was obtained from the parents. The mothers of children were requested to sign the consent form and fill out a questionnaire regarding the presence of congenital anomalies, medication intake, cigarette smoking, tobacco use and intake of folate during the three months before and one month after conception and pregnancy.

In brief, 3 *ml* of peripheral blood was collected in tubes containing 200 *μl* of 0.5 M EDTA, and genomic DNA was extracted from the peripheral blood using the salting out method 24. Genotyping of the TGFA/BamHI (rs11466297), TGFA/RsaI (rs3732248), BMP4 (rs17563) and BMP2 (rs235768) was performed by Polymerase Chain Reaction (PCR) and Restriction Fragment Length Polymorphism (RFLP) methods, according to a previous study 24. A total volume of 25 *μl* containing 30 *ng* of genomic DNA, 10 *pmol* of each primer, 1 *μl* dNTPs mix (Fermentas, Life Science), 2.5 *μl* 10×buffer and 0.5 *U* of Taq DNA polymerase (Fermentas Life Science, Lithuania) with 1.5 *mM* MgCl_2_ was prepared in 0.5 *ml* Eppendorf microtube for amplification of the target sequences. Amplification conditions started with an initial denaturation step of 4 *min* at 95*°C*, followed by 33 cycles of 45 *s* of denaturation (94*°C*), 30 *s* of annealing (60*°C*) and 40 *s* of extension (72*°C*), ended by a final extension for 5 *min* (72*°C*) and finally cooling to 4*°C*. All PCR products were subjected to 8% polyacrylamide gel electrophoresis and stained with silver nitrate. Table 1 shows the primer sequences and the pattern of restriction fragments.

**Table 1.** Primer sequences, product size and RE fragments for the BamHI, RsaI, BMP4 and BMP2

**SNPs**	**Global MAF**	**Primer(5′→ 3)**	**Product Size (*bp*)**	**RE fragments (*bp*)**
**TGFA/ BamHI (rs11466297 A/C)**	C=0.0238	F: GCCTGGCTTATTTGGGGATT R: AAGGGCAAGGAAACACAGG	174	A allele=120+54 C allele=174
**TGFA/RsaI (rs3732248 C/T)**	A=0.2075	F: TGCCTTCCTTCTGCTATCACT R: CAGAGCCAATGTCACCAAGT	153	C allele=91+75 T allele=166
**BMP4(rs17563)**	C = 0.3257	F: CACCATTCATTGCCCAAC R:AGTTTGGCTGCTTCTCCC	179	AA: 165 TT: 86 + 79 AT: 165 + 86 + 79
**BMP2(rs235768)**	T = 0.2332	F: GAAACGAGTGGGAAAACAACC R: GAGACACCTTGTTTCTCCTCCA	165	TT: 118 + 61 CC: 179 TC: 179 + 118 + 61

### Statistical analysis

Data were analyzed using SPSS 18 (SPSS Inc., Chicago, USA). Chi square and Fisher’s exact test with Open Epi Version 2.2 (free statistical software) were performed to compare genotype and allele frequency among the study groups. Logistic regression was used to determine the effect of influential factors and the interaction effect of different variants on the occurrence of CLP. p<0.05 was considered statistically significant.

## Results

There were 59.3% males and 40.7% females in the case and 43.6% males and 56.4% females in the control group. The results showed that gender had no significant association with the occurrence of CLP (p= 0.335).

Table 2 shows the genotype and allele frequency of TGFα variants namely Rsal and BamHI in the case and control groups. Table 3 shows the genotype and allele frequency of BMP2 and BMP4 in the two groups. Table 4 demonstrates the interaction effect of Rsal, Bam-HI, BMP2 and BMP4 polymorphisms on the occurrence of NSCLP. Multiple logistic regression showed that the interaction effect of polymorphisms of the BMP family, TGFα family and combination of all four on the occurrence of NSCLP was not statistically significant (p=1.000).

**Table 2.** Genotype and allele frequency of the TGFα BamHI and RsaI polymorphisms in the case and control groups

**Genotype/allele**	**Case group**	**Control group**	**p-value**	**OR (95% CI)**
**BamHI (rs11466297)**				
GG	97 (93.3%)	185 (92%)	Reference group	
GC	5 (4.8%)	16 (8%)	0.322	0.6 (0.2–1.7)
CC	2 (1.9%)	0(0%)	0.241	Undefined
G	199 (95.7%)	386 (96%)	Reference allele	
C	9 (4.3%)	16 (4%)	0.838	1.1 (0.47–2.5)
**RsaI (rs3732288)**				
CC	70 (66%)	112 (58.3%)	Reference group	
CT	30 (28.3%)	60 (31.3%)	0.409	0.8 (0.5–1.4)
TT	6 (5.7%)	20 (10.4%)	0.127	0.5 (0.2–1.3)
C	170 (80.2%)	284 (74%)	Reference allele	
T	42 (19.8%)	100 (26%)	0.087	0.7 (0.5–1.05)

**Table 3.** The genotype and allele frequency of the BMP2 and BMP4 polymorphisms in the case and control groups

**Genotype/allele**	**Case group**	**Control group**	**p-value**	**OR (95% CI)**
**BMP2 (rs235768)**				
TT	35 (32.1%)	124 (59.3%)	Reference group	
TA	65 (59.6%)	74 (39.4%)	0.561	0.2 (0.13–3)
AA	9 (8.3%)	11(5.3%)	0.002	2.7 (1.4–5.1)
T	135 (61.9%)	322 (77%)	Reference allele	
A	83 (38.1%)	96 (23%)	0.001	2 (1.4–2.9)
**BMP4 (rs17563)**				
TT	22 (20.2%)	74 (35.4%)	Reference group	
TC	70 (64.2%)	96 (45.9%)	0.585	1.3 (0.5–3].4)
CC	17 (15.6%)	39 (18.7%)	0.056	2.1 (0.98–4.5)
T	114 (52.3%)	244 (58.4%)	Reference allele	
C	104 (47.7%)	174 (41.6%)	0.142	1.3 (0.9–1.8)

**Table 4.** Interaction effect of BamHI, RsaI, BMP4 and BMP2 polymorphisms on the occurrence of NSCLP

**Interaction terms**	**p-value**
BMP2 c.893 T>A(rs235768) & BMP4 c.538 C>T (rs17563)	0.997
BMP4 c.538 C>T (rs17563) & TGFα (BamHI)	1.000
BMP4 c.538 C>T (rs17563) & TGFα (RsaI) rs3732248	1.000
BMP2 c.893 T>A(rs235768) & TGFα (BamHI)	1.000
BMP2 c.893 T>A(rs235768) & TGFα (RsaI) rs3732248	1.000
TGFα (BamHI) & TGFα (RsaI) rs3732248	1.000
BMP2 c.893 T>A (rs235768) & BMP4 c.538 C>T (rs17563) & TGFα (RsaI) rs3732248	1.000
BMP2 c.893 T>A(rs235768) & TGFα (BamHI) & TGFα (RsaI) rs3732248	1.000
BMP2 c.893 T>A(rs235768) & BMP4 c.538 C>T (rs17563) & TGFα (BamHI) & TGFα (RsaI) rs3732248	1.000

## Discussion

NSCLP is a common genetic defect that occurs due to the interaction of different genes and environmental factors 25. Several genes have been implicated in the occurrence of this defect among which *FGF8*, *FGFR1*, *MSX1*, *BMP* and *TGFα* can be named 26,27.

BMP4 and BMP2 are the main two subgroups of BMP superfamily, which play an important role in regulation of osteogenesis and chondrogenesis. They are expressed in epithelial and mesenchymal cells in the palatal shelves during palatogenesis 12. RsaI and Bam-HI are the two common variants of TGFα that induce extracellular matrix synthesis and migration of mesen-chymal cells. They also play a pivotal role in strengthening of the palatal tissue 8,9. Several studies have evaluated the relationship of the aforementioned genes and occurrence of CLP. The results of previous studies are controversial, which may be attributed to the difference in sample size, genetic background and environmental factors. Ebadifar *et al* showed that a correlation exists between polymorphism of BamHI variant and occurrence of CLP in the Iranian population such that the frequency of AC genotype and C allele was significantly higher in the patient group (12.4 and 8%, respectively) 11. Ardinger *et al* found a significant correlation between the polymorphism of BamHI and Tag 1 variants and the occurrence of CLP in the American population 8. On the other hand, Lidral *et al* found no significant correlation between *TGFα* gene and the occurrence of CLP in a non-Caucasian population 28.

Regarding *BMP* gene, Saket *et al* showed that the frequency of BMP4 and BMP2 genotypes was 70 and 59.8% higher in CLP patients compared to the values in the control group, respectively; this indicates a positive correlation between variants of this gene and occurrence of CLP in the Iranian population 15. Jianyan *et al* indicated that the frequency of BMP4 polymorphism was twice the value in the control group in patients with CLP in a Chinese population 29. On the other hand, Wang *et al* did not find a significant association between this polymorphism and occurrence of CLP in a Chinese population 30. Hu *et al*, in their meta-analysis stated that BMP4 increases the risk of occurrence of CLP in the Chinese population while it has a positive inhibitory effect on the occurrence of CLP in the Brazilian population 31.

The role of BMP4, BMP2, RsaI and BamHI polymorphisms in the occurrence of CLP in the Iranian population has been previously confirmed 11,15. Thus, this study assessed the interaction effect of these polymorphisms on the occurrence of CLP. The results showed that the interaction effect of two polymorphisms of *BMP* gene and *TGFα* gene had no significant effect on the occurrence of NSCLP. On the other hand, the overall and pairwise interaction effects of the four variants on the occurrence of CLP were not significant either.

Some previous studies have shown the interaction effect of different variants on the occurrence of NSC-LP. Song *et al* showed that combinations of rs2073485 (GG) and rs17176643 (aC+CC), rs2235371 (CC) and rs17176643 (aC+CC) and also rs2236909 (aG+aa) and rs17176643 (aC+CC) were significantly correlated with the occurrence of NSCLP 17. Sull *et al* demonstrated that the interaction of TGFα and IRF6 may play a role in the occurrence of NSCLP 32. Difference between the results of aforementioned studies and ours may be attributed to different study populations and the tested variants.

## Conclusion

Although the individual role of BMP4, BMP2, RsaI and BamHI variants has been shown in the occurrence of NSCLP separately in various studies in Iranian population 11,15, this study showed that their interaction effect on the occurrence of NSCLP was not significant.
